# Neuronal Representations of Tactic-Based Sensorimotor Transformations in the Primate Medial Prefrontal, Presupplementary, and Supplementary Motor Areas: A Comparative Study

**DOI:** 10.3389/fnsys.2020.536246

**Published:** 2020-09-30

**Authors:** Muhammad Ali Haider Awan, Hajime Mushiake, Yoshiya Matsuzaka

**Affiliations:** ^1^Laboratory of System Neuroscience, Department of Physiology, Tohoku University, Sendai, Japan; ^2^Division of Neuroscience, Faculty of Medicine, Tohoku Medical and Pharmaceutical University, Sendai, Japan

**Keywords:** supplementary motor area, presupplementary motor area, behavioral tactics, action selection, posterior medial prefrontal cortex

## Abstract

Adaptive context-dependent behaviors necessitate the flexible selection of multiple behavioral tactics, i.e., internal protocols for selecting an action. Previous primate studies have shown that the posterior medial prefrontal cortex (pmPFC) contributes to the selection, retention, and use of tactics, but the manner in which this area employs selected tactics to convert sensory information into action and how that manner differs from downstream cortical motor areas have yet to be fully elucidated. To address this issue, the present study recorded neuronal activity in two monkeys as they performed a two-choice arm reaching task that required the selection of multiple tactics when converting spatial cue information into the direction of arm reaching. Neuronal populations in both pmPFC and presupplementary motor area (pre-SMA) represented tactics during their selection, maintenance in memory, and their use in determining an action. Additionally, they represented the monkeys’ action in the behavioral epoch in which the direction of reaching was determined. A striking contrast between the pmPFC and the pre-SMA was the representation of the spatial cue location in the former and its absence in the latter area. In individual neurons, neurons in pmPFC and pre-SMA had either single or mixed representation of tactics and action. Some of the pmPFC neurons additionally encoded cue location. Finally, neurons in the supplementary motor area mainly represented the action. Taken together, the present results indicate that, of these three areas, the pmPFC plays a cardinal role during the integration of behavioral tactics and visuospatial information when selecting an action.

## Introduction

The executive function is to coordinate various cognitive processes to accomplish a particular goal in a flexible manner (Goldman-Rakic, 1998; Fuster, 2000; Funahashi, 2001). Damage to the prefrontal cortex (PFC) leads to inflexible behaviors in which subjects are not able to switch behavioral rules in a context-dependent manner (Eslinger and Grattan, 1993; Gershberg and Shimamura, 1995; Buckley et al., 2009; Schnyer et al., 2009). Neuroimaging studies in humans (Monchi et al., 2001; Mian et al., 2014) and neuron recording studies in animals (Hoshi et al., 2000; Wallis and Miller, 2003) have indicated that this cortical region plays a crucial role in switching among various rules to enable flexible behavior.

Recently, our research group found that neurons in the posterior medial PFC (pmPFC), which is located anterior to the presupplementary motor area (pre-SMA), exhibit prominent modulations in activity when a task required rapid selection of tactics that involved either reaching toward (pro-reach) or away from (anti-reach) a spatial cue (Matsuzaka et al., 2012). Surprisingly, this neuronal activity disappeared when the tactics were rendered invariant across trials, although the task still called for the selection of an action, which indicates that the pmPFC plays a crucial role in the selection of tactics rather than in the action *per se*. In contrast, neurons in the supplementary motor area (SMA) and pre-SMA continued to exhibit task-related activity modulation irrespective of whether the task required the monkeys to switch response tactics or simply follow a single tactic. A subsequent study showed that the pmPFC consists of separate neuronal populations that contributed to the inference of tactics from sensory cues, their retention in working memory, and their use to determine an action (Matsuzaka et al., 2016). Moreover, many of these neurons represent multiple types of information during the response period. In contrast, majority of SMA neurons were mostly selective for an action.

The fact that pmPFC neurons exhibited modulation of task-related activities only under the mixed-tactics condition in these previous studies is indicative of the pivotal role that this area plays when the protocol for deciding an action varies in an unpredictable manner. The former study (Matsuzaka et al., 2012) also showed that a considerable proportion of pre-SMA neurons represented the selected tactics, but it did not address the question whether these neurons played different roles from their counterparts in the pmPFC. The latter study (Matsuzaka et al., 2016) temporally separated the selection of tactics and action to clarify how the pmPFC contributes to the selection of tactics, their retention in working memory, and their use to determine an action. However, it did not compare the pre-SMA with either the pmPFC or the SMA under the same condition.

Thus, the present study aimed to determine whether there would be qualitative differences in neuronal activity among these three areas. Specifically, this study addressed the particular roles that these areas play in transforming visuospatial information into valid actions using the selected tactics. Under the pro-reach condition, the location of the spatial cue was concordant with the target of the reaching movement, whereas it was discordant with the target under the anti-reach condition. This difference necessitates the selection of tactics for sensorimotor transformation, the recognition of visuospatial information, and the conversion of that information into a valid action. The present results indicated that neurons in the pmPFC represented all types of information (i.e., tactics, cue position, and action), neurons in the adjacent pre-SMA represented tactics and action selection, and neurons in the SMA represented action selection.

## Materials and Methods

### Task Design

**Figure 1:**
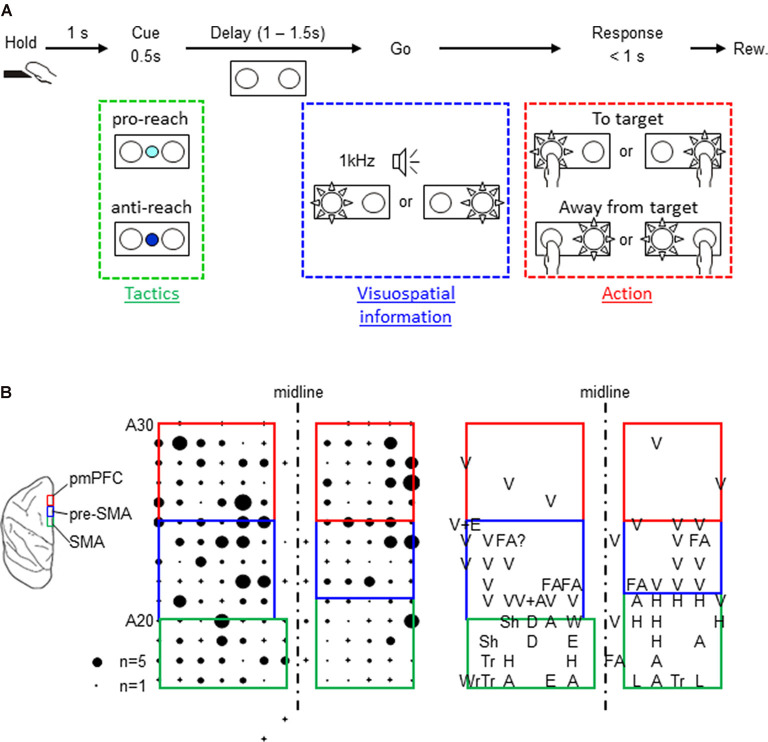
Task design and map of the three cortical areas. **(A)** Behavioral task. A trial started when the monkey pressed the hold button for 1 s, and the center color cue was turned on for 500 ms. The color cue instructed which tactics would be necessary for the subsequent reaching movement (cyan for pro-reach and blue for anti-reach). The cue was followed by random delay varying from 1 to 1.5 s. At the end of the delay, either the left or the right push button was back-illuminated by a white light-emitting diode, and the go signal (1 kHz beep tone) was simultaneously turned on, prompting the monkey’s response. The monkeys received a liquid reward for reaching toward (pro-reach) or away from (anti-reach) the appropriate illuminated button. Both monkeys made correct responses in over 80% of the trials. One monkey (monkey F) performed better in anti-reach trials though the reaction time was longer. The other monkey (monkey H) had an almost comparable performance between the two tasks (Supplementary Table 1). **(B)** Left: schematic illustration of the locations of the three cortical areas (posterior medial prefrontal cortex, presupplementary motor area, and supplementary motor area). Middle: distribution of one monkey’s task-related neurons. The size of the filled circles represents the numbers recorded in individual penetrations, and the cross signs represent the penetrations where no task-related neurons were recorded. Right: sensory response maps. A, arm; D, digits; E, elbow; FA, face; H, hand; L, leg; Sh, shoulder; Tr, trunk; Wr, wrist; and V, visual.

The present study included two Japanese monkeys (*Macaca fuscata*) that were cared for according to the guidelines of the National BioResource Project Japan and our Institute. During the experiment, the monkeys sat in a primate chair equipped with a hold button in the arm rest position while facing a panel that was equipped with two push buttons (one on the right and the other on the left side). The buttons on the panel were equipped with full-color light-emitting diodes (LEDs), and the panel also had an additional full-color LED in the center. The center LED was turned on in white to serve as a fixation target. To start a trial, the monkeys pressed the hold button. A trial began when the monkey pressed the hold button. After they kept pressing the button for 1 s, the center LED changed its color to either cyan or blue for 0.5 s to cue the tactics of the forthcoming arm reach. The cyan color required the reaching to be directed toward the subsequent spatial cue (pro-reach), whereas the blue color required the reach away from the spatial cue (anti-reach). Following a variable length of delay (1–1.5 s) that ensued the tactics cue, either the left or the right button was back-illuminated in white. At the same time, 1-kHz tone from a speaker was turned on as the go signal to prompt the monkeys to initiate the arm reach. The monkeys received liquid reward by reaching to the illuminated button in pro-reach trials within 1 s. In anti-reach trials, they were rewarded by reaching to the non-illuminated button. Both the spatial cue and the 1-kHz tone were turned off when the monkeys pressed either of the buttons or the 1-s time limit elapsed, whichever occurred earlier (Figure 1A). To select the appropriate action, the monkeys were required to select the relevant behavioral tactics (pro- or anti-reach) and then integrate them with the location of the spatial cue.

Throughout a trial, eye position was continuously monitored by an infrared eye monitor. The monkeys were required to keep the gaze on the center fixation point until the reward delivery.

### Neuronal Recordings

Neuronal activity were recorded from the medial region of the brain, including the pmPFC, the pre-SMA, and the SMA (Figure 1B), using glass-coated Elgiloy electrodes. The microelectrodes were advanced using hydraulic micromanipulators, and intracortical microstimulation (ICMS) was performed to measure the evoked movements. Additionally, the sensory responses of the neurons were examined by moving objects within the monkey’s visual field, manipulating the monkey’s joints, and touching the monkey to identify the cortical areas where the neurons were recorded (Figure 1B). The three cortical areas were identified using the anatomical and the physiological criteria reported previously (Matsuzaka et al., 1992, 2012). The SMA was identified as a dorsomedial frontal area where neurons were responsive to somatosensory but not to visual stimuli. Their somatosensory receptive fields were topographically organized and ICMS evoked bodily movements. The pre-SMA was located rostrally to the SMA. Contrary to the SMA, neurons in this region were unresponsive to tactile stimuli, but they responded to visual stimuli presented outside of the behavioral task. Movements were only occasionally evoked by strong ICMS in this region. Finally, the pmPFC was located rostrally and adjacent to the pre-SMA. In this region, neurons were responsive neither to tactile nor visual stimuli. No bodily or ocular movements were evoked by ICMS (up to 80 μA × 44 pulses).

### Statistical Analysis

#### Neuronal Database

The neurons in the present study include those used in our previous study (Matsuzaka et al., 2016) and the newly added neurons afterwards. Of the neurons recorded in the two monkeys (monkey F: 53 from pmPFC, 44 from pre-SMA, and 34 from SMA; monkey H: 176 from pmPFC, 105 from pre-SMA, and 80 from SMA), 153 pmPFC neurons, 113 pre-SMA neurons, and 73 SMA neurons were selected for the present analyses. The selection of neurons was made based on the criterion of sufficient correct trials (≥5); only trials in which the monkeys did not make an error were selected.

#### Neural Representation of Behavioral Factors by Individual Neurons

To examine the temporal variance of neural selectivity for tactics, action, and cue position, a moving time window (width: 200 ms; step: 20 ms) was used to calculate the instantaneous firing rate (IFR) in each trial, and the IFR for each neuron was then quantitatively analyzed to determine its dependence on tactics, action, and cue position using the following linear regression model:

IFR(t)=a1(t)×tactics+a2(t)×action+a3(t)×cue position+b(t)+ε(t),

where IFR(*t*) is instantaneous firing within the moving time window at time* t*, tactics is either pro- or anti-reach, action is left or right arm reach movement, cue position is the illuminated button (either left or right), a1(*t*), a2(*t*), and a3(*t*) are the respective regression coefficients, *b*(*t*) is the intercept, and ε(*t*) is the residual error at time *t*. As an indicator of neuronal selectivity, the coefficient of partial determination (CPD), which is the percentage of the neuronal firing rate variance that can be explained by factor *X*, was computed as follows:

$${\text{CPD}}_{\left(X,t\right)}=({\text{SSE}}_{\text{partial}(t)}-{\text{SSE}}_{\text{full}(t)})/{\text{SSE}}_{\text{partial}(t)}$$

where CPD (*X*, *t*) is the value of CPD at time *t*, SSE_partial(*t*)_ is the sum of squared errors when factor *X* is omitted from the above mentioned linear regression model, and SSE_Full(*t*)_ is the sum of squared errors when all factors are present. We calculated the CPDs of the three signals (tactics, cue position, and action) individually by removing the corresponding term from the above mentioned regression model (see Supplementary Text 1).

#### Population-Level Analysis of Representation of Tactics, Action, and Cue Position

The population mean value of CPD for behavioral factor *X* at time *t*, CPD_mean_(*X*, *t*), was calculated by averaging the CPD of the individual neurons in the neuronal population in each cortical area. Then, the deviation, ΔCPD_mean_(*X*, *t*), from the baseline period was calculated as follows:

$$\Delta {\text{CPD}}_{\text{mean}}(X,t)={\text{CPD}}_{\text{mean}}(X,t)-{\text{CPD}}_{\text{base}}(X)$$

where CPD_base_(*X*) is the mean CPD value for *X* during the pre-cue baseline period. To determine whether the neuronal selectivity values for tactics, action, and cue position during the delay and the response periods significantly differed from those in the pre-cue baseline period, a permutation test was performed. First, surrogate data sets were generated by randomly swapping the CPD values of individual neurons in the baseline period with the CPD values in a particular time window (*t*). Then, to compute the hypothetical distribution of ΔCPD_mean_(*X*, *t*) based on the null hypothesis that CPD_mean_(*X*, *t*) and CPD(*X*) during the baseline period belong to the same distribution, the surrogate data were used to compute the difference in CPD between time *t and* baseline period, i.e., Δ′CPD_mean_(*X*, *t*). These processes were repeated 10,000 times. The ΔCPD_mean_(*X*, *t*) was considered significant if it was outside the 99% confidence interval of the distribution of Δ′CPD_mean_(*X*, *t*).

## Results

**Figure 2:**
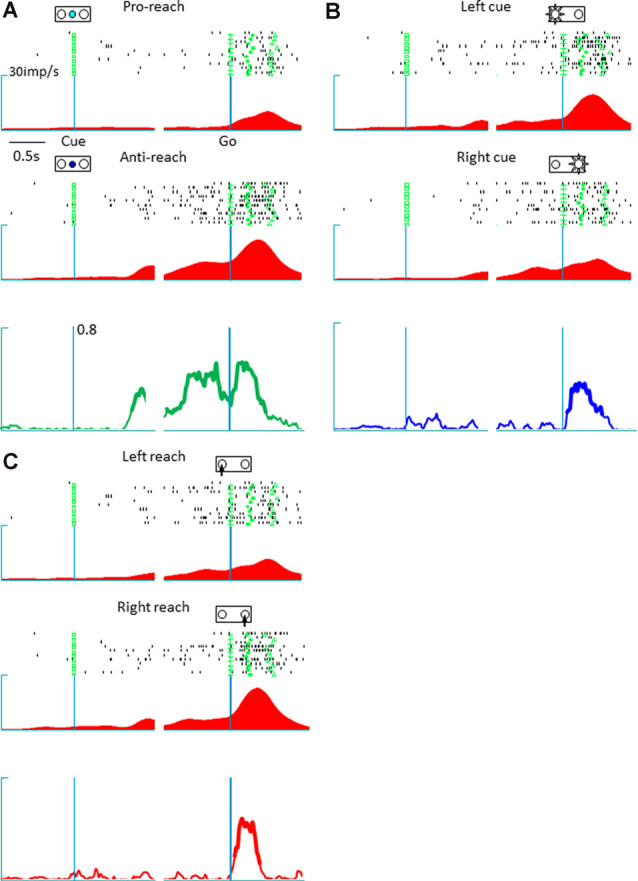
A representative posterior medial prefrontal cortex neuron in which the activity exhibited mixed selectivity for tactics, action, and cue position. **(A)** Raster display and spike density function of tactic-selective neuronal activity. The trials are divided according to tactics, irrespective of cue location and reach direction; the green squares, crosses, circles, and triangles represent the cue onset, go signal onset, hold button release, and target button press, respectively. Top: activity during a pro-reach trial. Middle: activity during an anti-reach trial that shows tactic selectivity in the representative posterior medial prefrontal cortex neuron. The Raster displays and spike density functions are aligned with the cue onset (left) and Go onset signal (right); the abscissa represents time, and the ordinate represents spike density function. This neuron exhibited preferential activation under the anti-reach condition during the latter half of the delay and the response periods. Bottom: temporal profile of tactic selectivity illustrated as time-resolved change in the coefficient of partial determination (CPD) value for tactics; the thickness of the line indicates significant dependence of IFR(*t*) on the tactics in a multiple-regression model using tactics, action, and cue position as regressors (*p* < 0.01). **(B)** Cue position-selective activity. The trials are grouped according to cue location (top, left cue position; middle, right cue position). The CPD for cue position is shown by a blue line (bottom). This neuron exhibited enhanced activity during the response period when the spatial cue appeared on the left. **(C)** Action-selective activity. The trials are grouped according to reach direction (top, left reach; middle, right reach). This neuron preferred reaching movements to the right. The CPD for cue position is shown by a red line (bottom).

**Figure 3:**
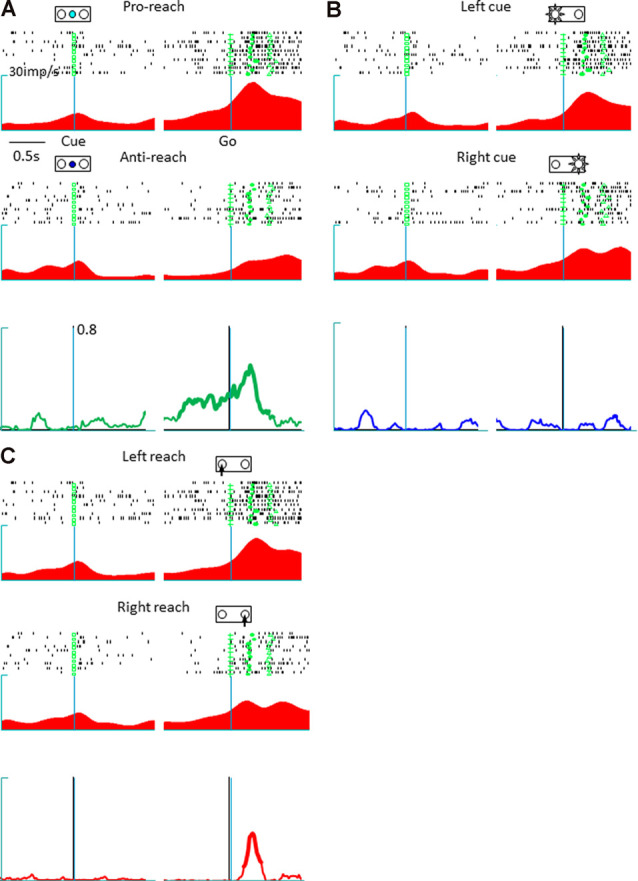
A representative tactic- and action-selective presupplementary motor area neuron; the legends are the same as in Figure 2. **(A)** Tactic-selective activity. This neuron showed selectivity for the pro-reach condition during the delay and the response periods. **(B)** Cue position-selective activity; this neuronal activity was non-selective for the spatial location of the cue. **(C)** Action-selective activity. This neuron was selective for left reaching movements.

The present study involved a quantitative analysis of neuronal activities in the pmPFC (*n* = 153), pre-SMA (*n* = 113), and SMA (*n* = 73) in terms of their selectivity for tactics, action selection, and cue position. The pmPFC exhibited selective activities for tactics, action, and cue position. Figure 2 shows the activity of a representative pmPFC neuron and its selectivity in terms of tactics (anti-reach), cue position (left cue position), and action (right movement). This neuron was selective for anti-reach (away from the target) during both the delay (before the go signal) and the response (after the go signal) periods. Figure 2A shows the preferential activation of this pmPFC neuron during anti-reach trials that began during the delay period and continued through the response period. In the response period, the same neuron exhibited selectivity for the location of the spatial cue (Figure 2B) and for action (Figure 2C). In contrast, the pre-SMA was selective for tactics and action but not for cue position. Figure 3 shows a representative pre-SMA neuron that was selective for tactics (pro-reach) and action (right movement), but not for cue position. Neurons in the SMA were mainly action selective.

**Figure 4:**
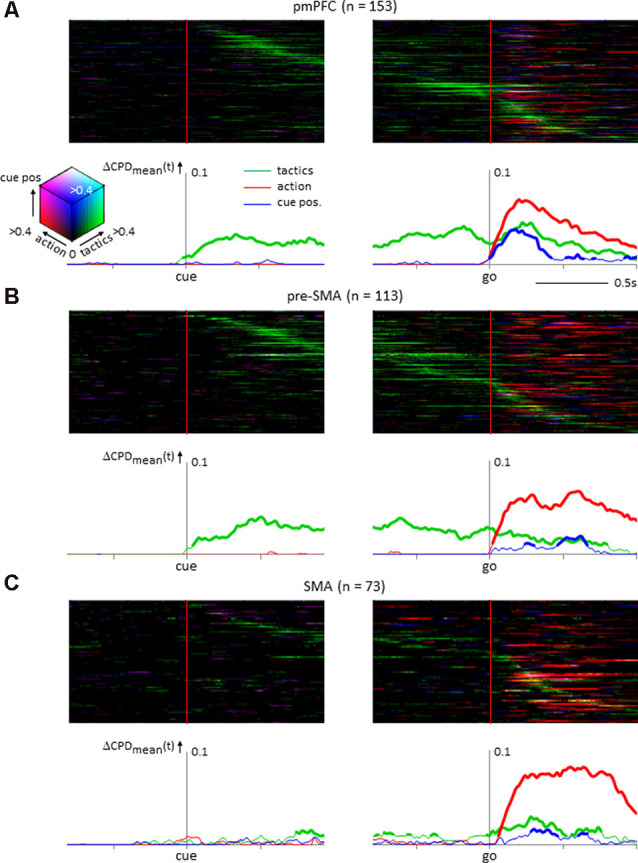
Temporal variance of neuronal selectivity for tactics, action, and cue position calculated from the instantaneous firing rate of neurons (see “Materials and Methods” section for details). **(A)** Posterior medial prefrontal cortex neuronal population (*n* = 153). In the upper half of the figure, the individual neuron’s coefficient of partial determination (CPD) values are illustrated as color-coded matrix. Each horizontal line represents a single neuron’s activity aligned with the onset of the tactics cue (left) and the go signal (right). The neurons are sorted by the timing of the peak CPD value for tactics. The CPDs of tactics, action, and cue positions are coded in green, red, and blue, respectively. The compound strength of selectivity is coded by the brightness of the respective color. The line graphs below illustrate the mean CPD value averaged across the population. The ordinate is the dCPD (increase of the mean CPD from the baseline period). The thickness of the lines represents a significant increase from the baseline period (permutation test, *p* < 0.01). **(B)** Neuronal population from presupplementary motor area (*n* = 113) showing selectivity for tactics and action during the delay and the response periods; cue position selectivity was not prominent. **(C)** Supplementary motor area (SMA) neuronal population (*n* = 73) showing action selectivity during the response period.

To investigate how individual neurons sampled from the pmPFC, the pre-SMA, and the SMA encoded multiple behavioral factors, time-resolved computations of the CPD values for tactics, action, and cue position were conducted (Figure 4). The pmPFC exhibited tactic-, action- and cue position-selective activities. Additionally, the tactic selectivity showed a different timing among the individual pmPFC neurons, whereas the selective activity for action and cue position appeared only in the response period in which this information was made available to the monkeys (Figure 4A). In the adjacent pre-SMA area, tactic selectivity was prominent during the delay and the response periods, and the timing of the selectivity varied among neurons, whereas action-related activity occurred during the response period; cue position selectivity was not significant in the pre-SMA (Figure 4B). The SMA exhibited strong action selectivity during the response period that started with the onset of the go signal and continued through the response period. A minority of SMA neurons exhibited strong selectivity for tactics during the delay. However, at the population level, the tactic representation by SMA neurons did not exhibit as strong and continuous elevation as in those by pmPFC and pre-SMA ones. Finally, the selectivity for cue position was not significant during the response period (Figure 4C).

## Discussion

The present study found that neuronal populations in three medial frontal cortical areas were involved in different processes of sensorimotor transformation when using multiple tactics. The pmPFC neuronal population encoded tactics, spatial information about the visual cue, and the resultant action. Neurons in the pre-SMA represented tactics and action, and neurons in the SMA mostly represented action. Extending those findings, the present study indicated that the pmPFC also encoded relevant sensory information (i.e., cue location) and that this information was absent in the pre-SMA and the SMA.

Anatomical studies have indicated that the primate PFC receives afferent projections from higher-order sensory association cortices in which peripheral sensory information is integrated to reconstruct internal representations of the behavioral context (Rao et al., 1997; Fuster, 2000, 2015; Wallis and Miller, 2003). Additionally, the PFC is one component of a supervisory attentional system (Norman and Shallice, 1986) that contributes to the evaluation and the selection of information relevant to the guidance of purposeful behaviors (Stuss, 2011). Consistent with these previous findings, the present results showed that neuronal representations of tactics, action, and cue position were present in the pmPFC. Efferent projections from the PFC are directed to cortical motor areas, particularly rostral motor areas such as the pre-SMA, rostral premotor areas, and rostral cingulate motor area (Tanji and Hoshi, 2008). In contrast, caudal motor areas, including the SMA, receive few, if any, projections from the PFC, which instead heavily project to the primary motor cortex and the spinal cord (Luppino et al., 1993; Dum and Strick, 1996). Thus, the presence of tactic-related representations in the pre-SMA and the predominance of action representation in the SMA likely reflected their distinct afferent and efferent projection systems.

A notable difference between the pmPFC and the pre-SMA observed in the present study was the presence of cue position representations in the pmPFC and their absence in the pre-SMA (Figure 4). Previous studies have shown that neurons in the pre-SMA exhibit spatially tuned activities during arm reaching movements done either in prescribed orders (Nakamura et al., 1998; Akkal et al., 2002) or to a chosen target (Hoshi and Tanji, 2004). Furthermore, anatomical studies found that the pre-SMA receives an abundance of afferents from the PFC (Luppino et al., 1990, 1993), a region where neurons exhibit spatially tuned visual responses (Goldman-Rakic, 1995). The present finding that pre-SMA neurons have little visuospatial information representation seems to contradict these past studies.

This discrepancy would be ascribable to the requirement for the selection of tactics and the involvement of the pmPFC in our study. In previous studies, the protocols to select appropriate actions were invariant even though the task called for the selection and the execution of actions on trial-by-trial basis. Under such condition, the pmPFC would not participate in the regulation of voluntary behavior (Matsuzaka et al., 2012). The involvement of the pmPFC in tactic-based sensorimotor transformation in the present study may have relieved the pre-SMA of the need to process the spatial information provided by the visual cue. The anatomical relationships between the parietal association cortex and the frontal cortex would be relevant to this interpretation. Although the pre-SMA receives dense projections from the PFC, direct projections from the parietal association cortex to the pre-SMA are sparse (Luppino et al., 1993). This finding suggests that spatial information sent to the pre-SMA is gated by the PFC. In support of this interpretation would be the view that pre-SMA function is dynamically controlled by the PFC (Picazio et al., 2014). It is also noteworthy that the hypoactivity in the PFC of schizophrenic patients is accompanied by increased compensatory activity in the pre-SMA, which suggests that normal functioning in the PFC represses downstream motor areas in healthy brains (Cieslik et al., 2015).

In addition to the difference between the pmPFC and the pre-SMA, the present study also demonstrated a striking similarity between these areas. We found tactic information not only in pmPFC but also in pre-SMA. One interpretation of our findings is that pre-SMA might be involved in encoding and maintaining tactics but not in utilizing tactics, and pmPFC plays a supervisory role over pre-SMA. pmPFC might be involved in the “dynamic monitoring” of tactics. If multiple tactics are involved in behavior, pmPFC would play a supervisory role and integrate all relevant sensory information, including cue position. Pre-SMA would then be “unburdened” from integration of tactics and cue position (Koechlin and Summerfield, 2007). Pre-SMA still plays an important role in implementing action by following determination of action in pmPFC. Finally, SMA is recruited in the execution of action with pre-SMA. The finding that some SMA neurons represented tactics by their activity (Figure 4C) is consistent with our previous report (Matsuzaka et al., 2012), but such neurons were not prevalent. Consequently, at the population level, SMA failed to retain tactic information until the response like in pmPFC and pre-SMA. Once action execution starts, pmPFC would cease to supervise and control is shifted to lower motor areas. In our previous study, we found that the dynamic alterations of action selectivity in SMA depend on the demand for tactics (Matsuzaka et al., 2013). Following this line of interpretation, pre-SMA shows dynamic alterations of visual cue selectivity depending on the demand for tactics. Based on current and previous findings, we call this process as “dynamic supervisory control” by a hierarchically ordered shift of control from the rostral to the caudal medial frontal areas (Norman and Shallice, 1986). Although the current study indicates that tactic-guided sensorimotor transformation occurred in the medial frontal areas, how these areas interact with each other remains to be explored by further studies.

The medial prefrontal cortex of primates has been recently implicated for decision making and the evaluation of the outcome of one’s and other individual’s action under social context (Yoshida et al., 2011, 2012; Noritake et al., 2018). A quantitative comparison of the neurons in the pmPFC, the pre-SMA, and the SMA revealed that only the pmPFC contained a special group of neurons whose activity was predictive of other agent’s intention (Falcone et al., 2017). Regulation of voluntary behavior while interacting with other individuals requires flexible switching of protocol for action determination. The present study would shed light on a significant contribution of this area during social interaction when the tactics of behavior change dynamically.

## Data Availability Statement

The datasets generated for this study are available on request to the corresponding author.

## Ethics Statement

The studies involving animal subjects were reviewed and approved by The Institutional Animal Care and Use Committee of Center for Laboratory Animal Research of Tohoku University.

## Author Contributions

YM designed the study and collected the data. MA and YM performed data analysis and wrote the manuscript. HM provided data interpretations and helped with writing the manuscript. All authors contributed to the article and approved the submitted version.

## Conflict of Interest

The authors declare that the research was conducted in the absence of any commercial or financial relationships that could be construed as a potential conflict of interest.

## Supplementary Material

SUPPLEMENTARY FIGURE 1 A representative example of supplementary motor area neuron that exhibited action-selective activity during the response period. The legends are the same as in Figure 2. (**A–C**) The tactic-selective, cue position-selective, and action-selective activity, respectively. This neuron was preferentially activated with reaching to the right target.

SUPPLEMENTARY FIGURE 2 Number of neurons having significant selectivity for either tactics, cue position, action, or their combination during the response period. Their percentages are shown in parentheses. The response period was divided into three epochs of 300-ms width; epoch 1 following the cue onset, epoch 2 beginning 150 ms before and ending 150 ms after the hold release, and epoch 3 preceding the target hit. For each neuron, we selected the epoch that gave the highest firing rate. To examine neuronal selectivity for the behavioral factors, the neuronal firing rate during the selected period was analyzed using multiple-regression model:

firing rate=a1×tactics+a2×action+a3×cue+b+e

where tactic is the spatial concordance between the cue and the target (pro- or anti-reach), action is the direction of the subject’s arm reach, cue is the location of the visuospatial cue, a1, a2, and a3 are regression coefficients, b is the intercept, and e is the residual error. Neurons that showed significant effects (*p* < 0.05) of tactics, action, or cue position were regarded as selective for these factors.

SUPPLEMENTARY TABLE 1 Behavioral data of the monkeys. The correct rate, the reaction time, and the movement time (mean ± SD) are shown in the upper and the lower tables, respectively, separately for the tactics and the direction of reaching for each monkey.

SUPPLEMENTARY TEXT 1 In computing the coefficient of partial determination (CPD) of the three signals (tactics, cue position, and action), we treated them as independent signals because they do not have one-to-one correspondence between each other. Furthermore, we confirmed that the CPD of a particular behavioral factor was not equal to the CPD of the remaining two factors (e.g., CPDaction <> CPDtactics + cue_position). If one of the three signals (e.g., action) was dependent on the other two signals (e.g., tactics and cue position), then the residual error in the multiple-regression model would be explained by them. In that case, the CPD of action would be equal to that of cue position + tactics. Indeed the example neurons in Figure 2 and Figure 3 both have elevated CPD value of tactics and action during the response period, but only the former neuron has elevated CPD for cue position.
