# Supplementary material for: Neuronal Representations of Tactic-Based Sensorimotor Transformations in the Primate Medial Prefrontal, Presupplementary, and Supplementary Motor Areas: A Comparative Study
Source: Front Syst Neurosci. 2020 Sep 30;14:536246. doi: 10.3389/fnsys.2020.536246 (PMC7556293; doi:10.3389/fnsys.2020.536246)
Supplement: SUPPLEMENTARY FIGURE 1 — A representative example of supplementary motor area neuron that exhibited action-selective activity during the response period. The legends are the same as in Figure 2. (A–C) The tactic-selective, cue position-selective, and action-selective activity, respectively. This neuron was preferentially activated with reaching to the right target. [file Data_Sheet_1.PDF]

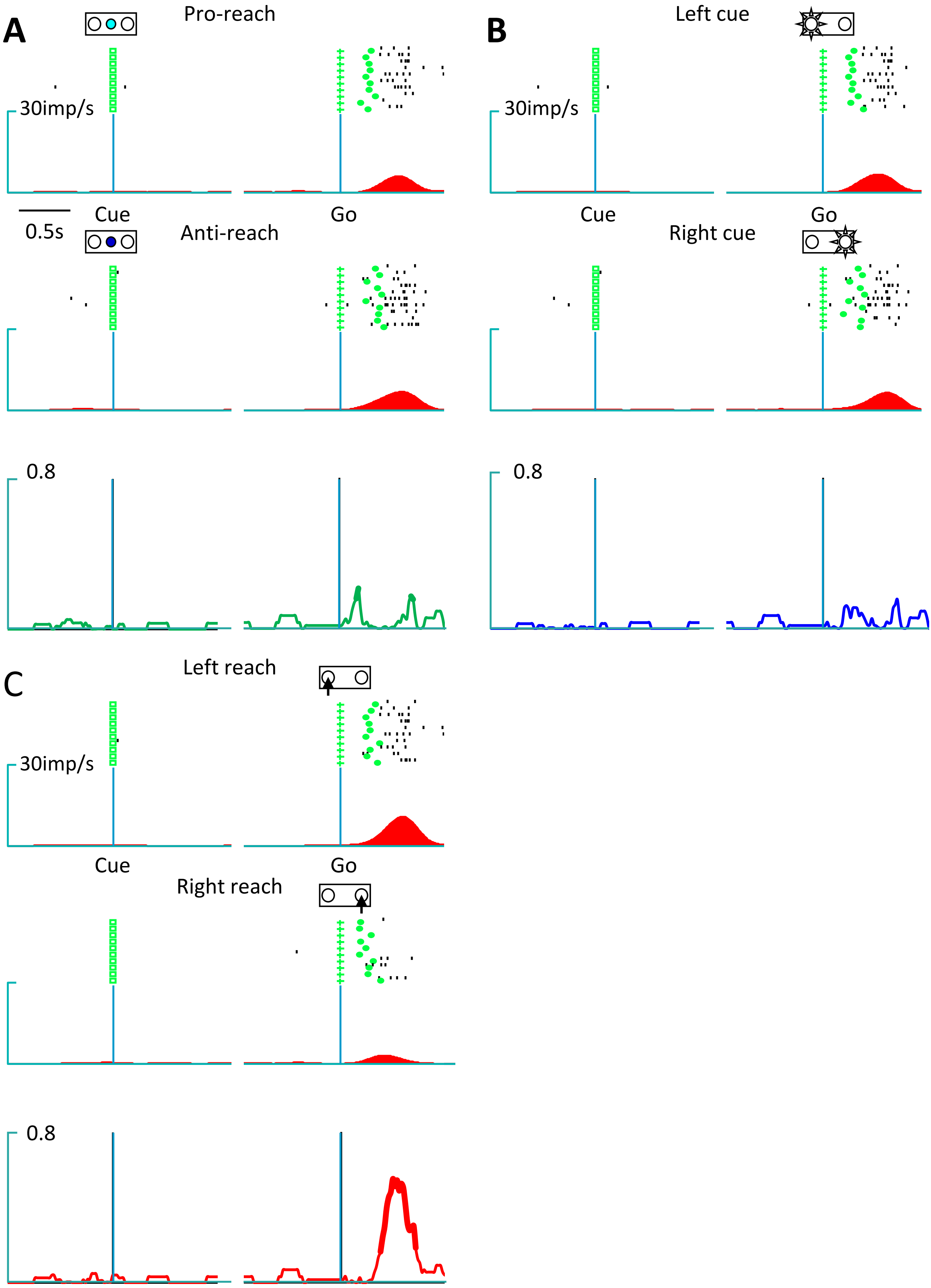

A
Pro-reach
30imp/s
0.5s
Cue
Go
Anti-reach
0.8
B
Left cue
30imp/s
Cue
Go
Right cue
0.8
C
Left reach
30imp/s
Cue
Go
Right reach
0.8

no. of neurons(percentage value)

pmPFC (n = 153)

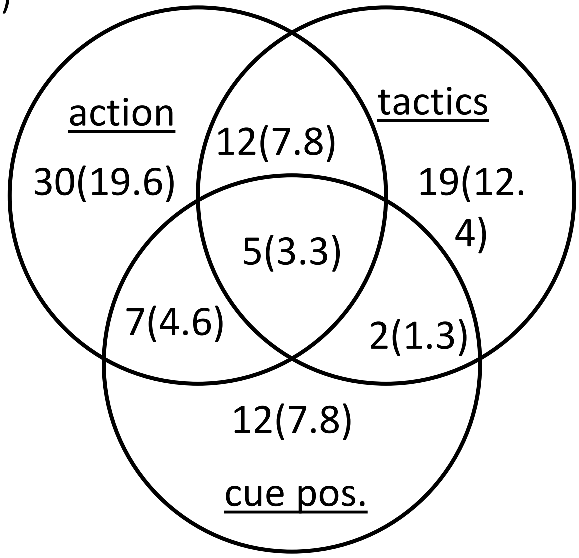

pre-SMA (n = 113)

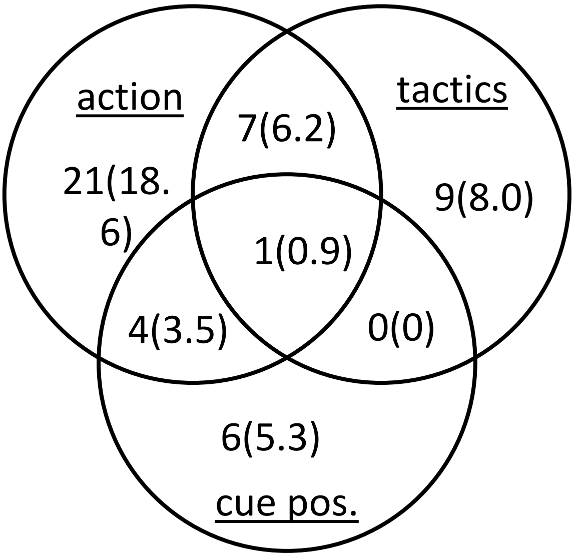

SMA (n = 73)

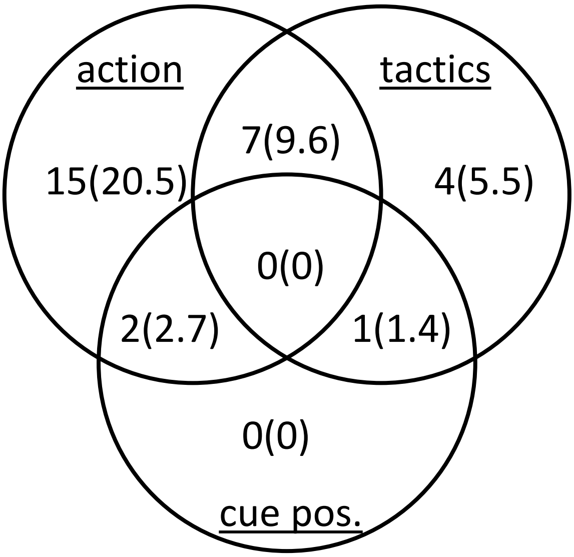

correct rate (%)

Monkey F		
	left	right
pro-reach	80.0%	84.9%
anti-reach	81.5%	88.5%

Monkey H		
	left	right
pro-reach	93.6%	94.4%
anti-reach	89.9%	92.4%

reaction time (ms)

	left	right
pro-reach	300.9±49.1	232.0±46.0
anti-reach	365.9±52.0	298.2±61.9

	left	right
pro-reach	295.6±58.1	269.0±49.3
anti-reach	294.1±58.9	264.1±52.3

movement time (ms)

	left	right
pro-reach	404.4±51.4	344.3±42.0
anti-reach	413.7±59.1	352.6±62.9

	left	right
pro-reach	296.1±54.4	323.2±51.5
anti-reach	300.1±49.8	276.0±46.4
